# Machine Learning for Renal Pathologies: An Updated Survey

**DOI:** 10.3390/s22134989

**Published:** 2022-07-01

**Authors:** Roberto Magherini, Elisa Mussi, Yary Volpe, Rocco Furferi, Francesco Buonamici, Michaela Servi

**Affiliations:** Department of Industrial Engineering, University of Florence, 50139 Florence, Italy; elisa.mussi@unifi.it (E.M.); yary.volpe@unifi.it (Y.V.); rocco.furferi@unifi.it (R.F.); francesco.buonamici@unifi.it (F.B.); michaela.servi@unifi.it (M.S.)

**Keywords:** renal pathology, deep learning, machine learning, artificial intelligence

## Abstract

Within the literature concerning modern machine learning techniques applied to the medical field, there is a growing interest in the application of these technologies to the nephrological area, especially regarding the study of renal pathologies, because they are very common and widespread in our society, afflicting a high percentage of the population and leading to various complications, up to death in some cases. For these reasons, the authors have considered it appropriate to collect, using one of the major bibliographic databases available, and analyze the studies carried out until February 2022 on the use of machine learning techniques in the nephrological field, grouping them according to the addressed pathologies: renal masses, acute kidney injury, chronic kidney disease, kidney stone, glomerular disease, kidney transplant, and others less widespread. Of a total of 224 studies, 59 were analyzed according to inclusion and exclusion criteria in this review, considering the method used and the type of data available. Based on the study conducted, it is possible to see a growing trend and interest in the use of machine learning applications in nephrology, becoming an additional tool for physicians, which can enable them to make more accurate and faster diagnoses, although there remains a major limitation given the difficulty in creating public databases that can be used by the scientific community to corroborate and eventually make a positive contribution in this area.

## 1. Introduction

Kidney diseases, such as renal tumors, acute kidney injury (AKI), and chronic kidney disease (CKD), are important issues for nephrology and public health worldwide, as they are associated with high mortality and morbidity rates [1,2]. These diseases, if not identified and treated preventively, can degenerate and lead to severe renal dysfunction, comorbidities, and, in the worst case, death [3,4,5]. Currently, in order to detect and prevent the degeneration of kidney disease, continuous monitoring of specific parameters obtained through diagnostic tests is performed [6]. Given that statistical models are used to determine the actual presence or absence of disease [7], its severity [8], or its degeneration [9], it is natural to think that models based on artificial intelligence (AI) and machine learning (ML) [10] could also be used to achieve this same goal, to obtain statistically better results or more high-performing solutions.

In the last decade, machine learning (ML) techniques have been increasingly employed in a variety of research areas. The consolidation of these methodologies, as well as the benefits of their employment, have occasionally made them the primary mode of operation in many sectors, such as object detection [11], speech recognition [12], emotion recognition [13], and sentiment analysis [14,15]. ML techniques have also captured the interest of the medical community, and multiple positive results have been achieved; some examples of healthcare applications are real-time prioritization and triage [16,17,18,19], personalized medications and care [20,21,22], and patient data analytics [23,24].

In nephrology, ML techniques are used for several purposes:

- segmentation and identification of the anatomy of interest within the diagnostic images (e.g., kidney masses such as tumors, cysts, etc.);
- classification of a kidney mass type, or of the stage in which a specific tumor is found;
- prediction of the evolution of kidney functionality, which can highlight the presence of pathologies.

Among others, ML techniques can be used in the analysis of suspicious renal masses. In such cases, it is nowadays necessary to surgically remove the tumor to identify if it is of a malignant or benign nature, but, due to its position, surgical removal is impossible without risking permanently compromising the patient’s urological function. For this reason, by working directly with diagnostic data and images, machine learning techniques can be crucial alternative solutions for segmenting and identifying masses.

Furthermore, some techniques can be used to help physicians to distinguish between particular cases of some pathologies that are very difficult to distinguish. In these cases, features obtained from diagnostic exams are used to classify the single cases; in this way, the physicians can reach a more precise diagnosis.

In addition to these applications, there are also techniques realized to prescribe specific therapies, or to detect a pathology in advance, in order to prevent it or any of the possible degenerative side effects (e.g., chronic kidney disease, acute kidney injury). In these applications are included also tasks with the aim to predict the compatibility and the outcome of a surgical operation, such as a kidney transplant.

Recently, the number of works related to this area has dramatically increased, rising from a few dozen papers before 2018, to a few hundred presently (based on papers indexed on the Scopus^®^ database from Elsevier). For this reason, it is crucial to carry out an updated survey summarizing the most promising opportunities offered by ML in this area. Accordingly, the present work aims to propose an updated and schematic survey of the most effective existing techniques and to draft possible future research lines based on ML.

First, the most promising articles are selected from the overall literature and classified based on their different applications. Then, in Section 4, there is a description and a comparison of all the used datasets relative to the works selected. Then, in Section 5, the implemented methods and the possible future developments are analyzed. Finally, in Section 6, conclusions are drafted.

The contribution that the authors intend to make with this work is to give a macroscopic view of the existing works concerning nephrology. In particular, the aim is to understand the state of the art of the methods that employ ML techniques to deal with some of the most common kidney diseases, reporting the various resulting metrics for each method. In addition, dimensional analysis of the various types of existing datasets that have been used so far is carried out and a generic comparison is made from the point of view of the type of data.

## 2. Article Selection

A study of the literature related to publications spanning from 1992 to February 2022 was carried out using Elsevier’s abstract and citation database, Scopus^®^, by entering keywords, “Artificial Intelligence”, “Machine Learning”, “Kidney”, to identify the most common and effective artificial intelligence (AI) and ML techniques that directly involve the kidney. In particular, the entered query was as follows:

TITLE-ABS (artificial OR intelligence OR machine OR learning OR kidney) AND  KEY (artificial AND intelligence AND machine AND learning AND kidney)(1)

**Figure 1:**
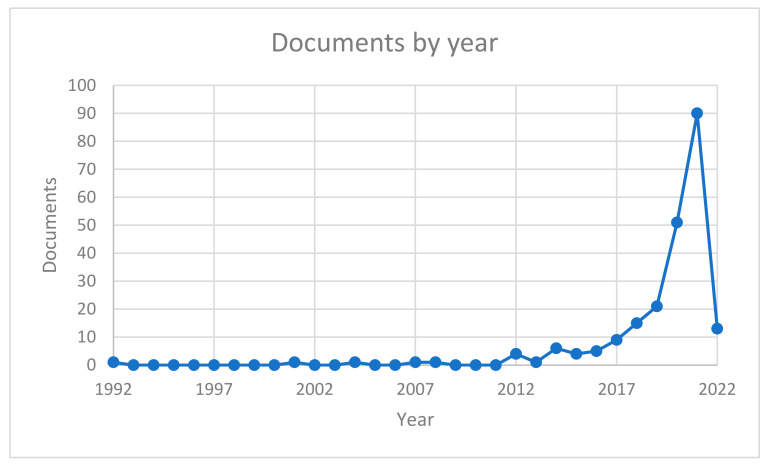
Trend of documents per year.

The research thus performed allowed the identification of papers that use AI and ML in kidney analysis contexts. Figure 1 shows a significant increase in recent years (after 2017) in the interest and production of papers by the scientific community—in general, there was an overall number of 224 papers dealing with the selected topic.

To focus on the most relevant works, the literature analysis was carried out according to the following inclusion and exclusion criteria.

Inclusion criteria: (1) articles dealing with ML and AI techniques applied to the kidney were considered; (2) original articles concerning one or more of the following aspects were taken into consideration—segmentation, classification, and prediction of diseases directly related to the kidney; (3) reviews related to these topics were studied to perform a final check of the selected articles.

Exclusion criteria: (1) editorials, commentaries, and abstracts were not included in this study; (2) studies related to animals or carried out only at a laboratory level were excluded; (3) research studies that were not applied in clinical practice were not considered.

According to the aforementioned procedure, fifty-nine studies were found to be eligible to be part of this survey.

## 3. Machine Learning Approaches for Nephrology

In the following, the studies are grouped based on the nature of the kidney disease. In detail, the analyzed pathologies are “kidney masses”, “acute kidney injury”, “chronic kidney disease”, “kidney stone”, “glomerular disease”, “kidney transplant”, and “other kidney pathologies”. From the analysis of the selected articles, three main research tasks are identified across the application areas:

(1) segmentation and identification, which intends to analyze diagnostic images with the purpose of highlighting or detecting one or more specific elements;
(2) classification, which aims to perform a diagnosis or to determine the degree of severity of disease;
(3) prediction, which aims to prevent or forecast some future event, e.g., predict either the degeneration of a disease or the outcome of a specific therapy.

**Figure 2:**
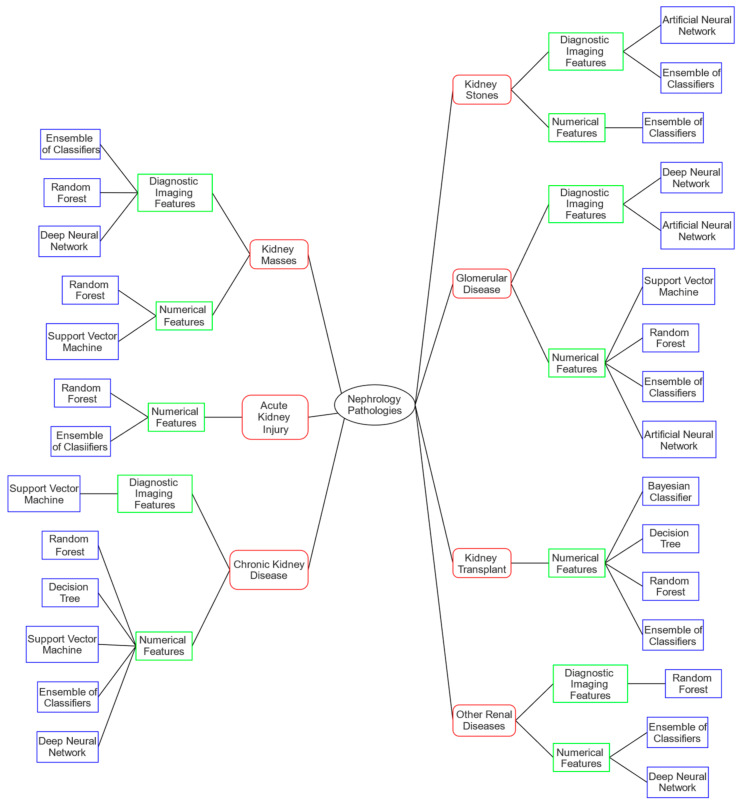
Scheme of pathologies with ML techniques applied according to the type of dataset available. Kidney disease addressed in red; type of available data in green; ML technique used in blue.

In the next subsections are reported, for each disease, a brief description of the symptoms to provide the reader with a simple explanation of the clinical scenario, and the various ML techniques used in the state of the art, grouped according to the research tasks described above, highlighting the type of database used. Figure 2 shows a graph schematically outlining the several analyzed pathologies (red color). From each pathology, one or two branches may be amplified according to the type of data available in the available studies (green color), and finally from these as many branches as the ML methods used on that type of data for that specific renal pathology (blue color). The following sections are based on the schematization depicted in the graph.

### 3.1. Kidney Masses

Kidney masses are abnormal growths within the kidney. They are mainly subdivided into two main categories: solid and cystic. Generally, the presence of a kidney mass is determined by relying on imaging techniques such as computed tomography (CT), magnetic resonance imaging (MRI), or ultrasound (US).

In general, cystic kidney masses are, in most cases, benign [25], while solid kidney masses are generally malignant; therefore, the kidney is generally partially or totally removed to perform the histological exam. However, approximately 16% of surgically removed solid kidney masses are benign [26] and surgical removal would not have been necessary. Unfortunately, the distinction of the nature of the solid renal mass, using diagnostic imaging, is very complex, even for specialized physicians, given the significant similarities in the appearance of some types of malignant and benign renal masses, in terms of texture, size, volume, and position. To face this challenge, modern ML techniques have been employed to process image data, proving to help physicians in making a more precise and accurate diagnosis. To classify and distinguish between malignant and benign masses, [27] some use a Bayesian classifier [28], a learning algorithm based on the statistical relationship between radiomics features (relational functional gradient boosting), and [29] an algorithm based on CT texture analysis. Many works focus on the analysis of renal cell carcinoma (RCC), which is the cause of 80% of kidney cancer deaths [25], either to distinguish different types of RCCs or to differentiate them from benign tumors. In [30,31,32,33,34], the main goal is to diagnose the most common malignant tumor, the clear cell RCC, using radiomic features and ML-based classifiers (e.g., random forest, CatBoost). Using radiomic features extracted from multiphoton microscopy images of kidney tissue sections, [35] try to distinguish RCC chromophobes and oncocytomas, while [36] try to classify the stage of a particular type of malignant tumor, the papillary RCC, using microarray datasets [37] and clinical information of the patients. Some more recent research, such as that of [38,39,40], focuses not only on tumor classification, but also on automatic tumor identification through diagnostic images, by using three-dimensional image processing with ML techniques such as 3D U-Net, and 3D V-Net; with these solutions, they are able to automatically segment the tumor inside the CT. Table 1 shows these works, explaining the main objective of each one, the adopted ML techniques, the database exploited, the best result achieved, and finally the year of publication; the reported metrics should be read from the perspective that the higher the reported value, the better the obtained performance.

### 3.2. Acute Kidney Injury

During an episode of acute kidney injury (AKI), the kidneys show difficulty in maintaining the proper fluid balance in the body, due to an accumulation of waste products. Given the speed with which it strikes and the damage that it causes, being able to detect it early can be of great significance. In this type of critical situation, AI is demonstrated to be one of the best solutions to correctly identify a patient with AKI. In studies by [45,46,47], the goal is to predict AKI based on early symptoms to prevent a possible degeneration of the disease, analyzing electronic health records (HER) and other clinical data, such as laboratory tests, vital signs, and patient demographics. AI techniques, thanks also to the speed of response, can be decisive, as in the case of [48], in which the authors try to detect AKI in burn patients using a k-Nearest Neighbor classifier on numerical features obtained from plasma creatinine testing [49].

Some research, such as [50,51], focuses on predicting an episode of AKI in patients undergoing examinations that require contrast agents, specifically coronary angiography. It has been observed that the use of such agents can lead to AKI episodes; in these studies, the authors aim to predict the AKI episode with AI approaches by using clinical variables collected before the examination and by the results of the coronary angiography that they undergo [52].

Recent studies focus on predicting AKI episodes’ insurgence within different periods from its manifestation. The most common prediction time intervals vary from 48 h to a maximum of 90 days, as in [53]; in this work, the authors evaluate their solution based on the analysis of time-series data over these time intervals. It is possible to find another example in [54], in which the authors, through numerical features extracted from multiple blood tests per single patient, attempt to predict AKI within 30 days from its manifestation. Finally, in [55], the authors, using daily collected patients’ clinical data, propose a particular type of deep learning algorithm, based on time series, which is able to predict AKI within 48 h from its occurrence, as well as classify the stage of the AKI disease if it is already present. In Table 2, analogously to Table 1, are reported all the related objectives, methods, used databases, and results.

### 3.3. Chronic Kidney Disease

Chronic kidney disease (CKD) is a condition characterized by the gradual loss of kidney function over time. CDK damages the kidneys by decreasing their ability to filter waste from the blood. In severe conditions, waste can reach high levels and lead to the development of other complications, which, in the most extreme cases, will require periodic medical treatment, such as dialysis, or even a kidney transplant [62]. CDK is a disease that can be diagnosed by physicians through the study and analysis of a variety of indices (e.g., eGFR [63]); thus, it is suitable for the application of ML methods. An example of using AI for this purpose can be seen in the study by [26], where the stage of pathology is classified using radiomic features obtained from ultrasound images of the kidney.

The general interest and applications to diagnose CKD underwent an abrupt increase with the creation and public release in 2015 of a database containing characteristic features (i.e., age, blood pressure, specific gravity, albumin, sugar, red blood cells, pus cell, pus cell clumps, bacteria, blood glucose random, blood urea, serum creatinine, sodium, potassium, hemoglobin, packed cell volume, white blood cell count, red blood cell count, hypertension, diabetes mellitus, coronary artery disease, appetite, pedal edema, and anemia) related to 400 patients during the early symptoms of the disease [64]. Different methods based on the analysis and classification of patient features are adopted by [26,65,66,67,68,69,70,71,72,73].

In addition to the diagnosis of CKD, there are some related studies in the literature, such as [74], in which the authors try to predict a possible plan for the patients’ diet, given the fact that following a proper and suitable diet plan can help to slow down the progress of CKD [75]. In [76], since maintaining appropriate hemoglobin levels during treatment for CKD is critical, the authors try to predict the hemoglobin level in the blood during anemia treatment in predialysis CKD patients, to intervene more quickly.

This information, the used databases, and the obtained accuracy results are shown in Table 3, analogously to the others.

### 3.4. Kidney Stone

Nephrolithiasis, or kidney stones, is a condition characterized by the presence of deposits in the kidney, caused by an alteration in the balance between the solubility and precipitation of salts in the urinary tract and kidneys [78]. One crucial point is given by the fact that surgery is required in 20% of patients with this condition [79]. In this context, AI is applied to identify the correct type of treatment to be followed based on parameters such as sediment composition, location, and size [80]. Some research focuses on the detection of kidney stones, such as [81,82], which use radiomic features extracted from manually segmented CT, with the goal of the early detection of stone deposits before they reach a size greater than 2 cm, allowing the use of non-invasive treatments. Other research, such as [83,84,85], focuses on predicting the outcome of shock wave treatment without the use of diagnostic imaging techniques, by analyzing the preoperative parameters of patients (such as age, sex, presence of related diseases, and stone characteristics including stone laterality, location, and maximum length). Similar to the other tables, Table 4 reports this information, the databases used, and the accuracy of the obtained results.

### 3.5. Glomerular Diseases

Glomerular diseases are diseases that affect the glomeruli, whose function is to filter blood and, at the same time, to retain proteins and blood that the body needs. Many diseases, such as diabetes, affect kidney function by attacking the glomeruli [86]. In this regard [87,88,89], use methods based on the analysis of patients’ clinical data to predict type II diabetes. Some studies focus on specific conditions and causes of glomerular diseases, such as Immunoglobulin A Nephropathy (IgAN), which is the most common biopsy-proven primary glomerulonephritis in the world [90]; it damages not only the kidneys, but also the immune system response [91]. In [92,93,94], the authors implement applications able to predict IgAN using a renal immunofluorescent image obtained by fluorescence microscopes relative to a renal biopsy. Other works, such as [95,96,97], focus on detecting type II diabetes directly from diagnostic images, using radiomic features. Finally, [98] try to predict the weight of children with glomerular disease to avoid possibly dangerous weight loss, using diagnostic numerical features obtained from blood monitoring and analysis.

All the useful information is reported in Table 5, analogously to the previous tables.

### 3.6. Kidney Transplant

Even if kidney transplantation is not a pathology but rather a specific surgical treatment, some authors considered creating a dedicated section since there are several studies regarding this topic, and it is one of the most common treatments for patients with severe kidney pathologies.

In detail, kidney transplantation is a surgical procedure that involves taking a healthy kidney from a living or cadaveric donor and implanting it into the recipient patient. For the transplant to be successful, many factors must be considered, including the compatibility of the donor with the human leukocyte antigen (HLA) proteins of the recipient. Although, nowadays, there is a method that reduces the risk of rejection, in the case of mismatched HLA [99,100], approximately 40% of donated kidneys are rejected [101]. The ML techniques applied by [102,103,104,105,106] focus on predicting the probability of success and survival in these types of interventions using numerical features (e.g., age, sex, time in dialysis, donor type, donor age, HLA mismatches, delayed graft function, acute rejection episode, and chronic allograft nephropathy). Table 6 reports all the necessary information, analogously to the others.

### 3.7. Other Renal Diseases

In this group are reported other renal diseases that do not fit within the classification provided so far. These studies focus on uncommon objectives, such as [108], which aims to predict the level of hemoglobin in patients with renal dysfunction, using numerical characteristics obtained from clinical data related to dialysis [109]; in [110], an application is developed that intends to define the need to perform or not a renal biopsy by analyzing physicians’ annotations through a natural language processing ML algorithm; [111] try to predict the survival of hemodialysis patients using numerical characteristics (age, sex, diabetes mellitus, chronic glomerulonephritis or nephrosclerosis, body mass index, albumin, sodium, potassium, calcium, phosphorus, creatinine, total cholesterol, etc.). In [112], the authors extract radiomics features from three-dimensional ultrasound images to identify renal and liver tissue in patients with hydronephrosis. Finally, [113] use numerical features extracted from patients’ EHRs with the corresponding acquisition time, to predict the risk of stratification of renal function deterioration.

Table 7 is presented analogously to the previous ones.

## 4. Databases Used in Reviewed Research

In this section, two tables contain information about the databases used in the research considered. This information includes the name of the database, when available, or otherwise a distinctive name related to the type of data and the organization in which they were collected; the number of elements that make up the dataset; a brief description of the type of data present; the year in which the database was made public, when available, otherwise the year in which it was used for the first time in a paper; and, finally, whether the database is open access.

Specifically, in Table 8 are reported all databases that have as the data type diagnostic images; this can be CT, MRI, US, or images obtained through analysis in the laboratory with instruments such as a digital microscope. This second type of technique is mainly used for the detection of masses or malformations within the kidneys. It is possible to note that these types of databases have very different volumes; in the case of 3D US images, there are, for example, databases of nine patients; for CT and MRI, there are databases with a minimum of 50 cases up to a few hundred, and finally, with regard to other imaging techniques, there are databases from a minimum of 24 up to a maximum of 1321 cases. This discordance at the numerical level is given mainly by the effectiveness and invasiveness of the different examinations and therefore by the frequency of their use in clinical practice. US is a less effective imaging technique in this field, compared to CT and MRI, and, therefore, the studies concerning the application of this technique are very small and dated. As for the examinations performed on biopsies, the number of samples is much larger because it is an examination that is compulsorily performed in every case to define with absolute certainty the type of mass removed. Among the reported databases, only two are publicly accessible: the CPTAC Clear Cell Renal Cell Carcinoma Discovery Study [41,114], released in 2018 by the U.S. National Cancer Institute, and kits2019 [44], released in 2019 by grand-challenge.org, hosted by MICCAI.

In Table 9 are reported all the databases exclusive of numerical type, relating to information obtained from diagnostic tests, such as blood tests, genetic tests of kidney tissues, or data from patient history. For these databases, the volume varies; for more complex tests, such as genetic tests, there is a variation ranging from a few tens up to a few hundred cases; for medical histories, this ranges from a few hundred up to 269,999 cases; for simpler diagnostic tests, from a few tens up to several thousand cases. Of these databases, only three are publicly available; for some, access is limited to a specific country (in the table, these are reported as “only in the USA”). Among the public databases, two contain RNA sequences of renal tumors, which are used to identify the pathological stage of the tumor. Finally, the third public database contains data on blood tests, patient history, and information about CDK-related diseases.

## 5. Discussion

After having reported in the previous sections the existing methods in the literature to address renal pathologies with machine learning methods and analyzed the available databases, we summarize in this section what has been found for each pathology; in particular, the limitations of the studies carried out so far and possible future developments will be indicated.

Regarding renal masses, the goal of the analyzed works is to find a method to non-invasively discriminate benign and malignant masses [29], and artificial intelligence has the potential to become a very important tool for assisted diagnosis. This is motivated by the results of identified research, in which are obtained accuracies ranging from 79% [32] to a peak of approximately 90% [29] (these results are from private single-center databases). Currently, the gold standard for the detection of a renal mass is based on the analysis, by an experienced physician, of CT images before and after dosing with a contrast medium [115]. AI can perform the discrimination function because it can analyze diagnostic images, such as CT, at a very high or equal level of detail as an expert [116]. This is because it can also take into account multidimensional characteristic features, such as texture. However, using CT, the various parameters used for the acquisition and the timing with which it is done assume an important role [29]. In fact, from the articles analyzed, it emerges that, according to the CT acquisition phase taken into consideration, the results obtained change; specifically, the most used phase is the corticomedullary phase [30]. Furthermore, as regards the use of CT for the extraction of characteristic features, the literature considers the three-dimensional use of CT to be better and more representative [117], but in the research identified [27,28,29,30,31,32,33,34,35,36], to reduce the workload of manual segmentation and facilitate the repeatability of this operation, a limited number of slices or only the two-dimensional slice containing the largest portion of the mass considered is used. In addition to how CT is used, it is also important to control the method by which features are extracted; in some research [28,29,31,32,33,34,35,36], radiomic features are used, after manual segmentation by at least one experienced physician, to classify tumors. One of the major limitations introduced, in doing so, is the bias of the operator who performs the segmentation [118]. For this reason, more recent studies [38,39,40] have focused on overcoming manual segmentation by creating deep learning algorithms capable of automatically segmenting kidneys and tumors present in CT; the results obtained from these studies are positive, as they achieve a mean kidney tumor size–mean per CT of the testing set of (Kidney Sørensen-Dice + Tumor Sørensen-Dice)/2 [44], with a maximum of 0.9168. In particular, one solution proposed in the literature to deal with operator-introduced bias is for a team of clinicians to collaborate on the kits2019 database in a way that reduces the risk of bias as much as possible.

Regarding AKI, this pathology is very widespread, with consequences that, if not treated in time, can even lead to death. Currently, there is no specific intervention that can prevent AKI; there are only general measures that can be taken to delay more critical procedures such as surgery [55]. For this reason, most of the recently developed research focuses on predicting the prognosis of this disease [45,46,47,48,50,51,53,54,55], being able to predict AKI with good accuracy even 30 days in advance [54]. The solutions implemented depend not only on the task but also on the actual number of data available for each patient [119]. Maintaining a large number of data for each patient has an economic cost and features used in one center may not be available in other centers [45]. ML techniques can outperform clinical tools used to estimate AKI risk, as we see in [46], with an AUC of 0.85. The performance of solutions exploiting ML for the prediction of AKI is positive: AUC 0.76 [47], in liver transplant patients; 97% accuracy [48] and AUC 0.76 [55], for burn patients; AUC [0.79–0.843] [50,51], for patients undergoing coronary angiography. However, despite the various existing applications, there is a lack of a ML-based prediction systems that can be recognized as state of the art for AKI prediction [47].

Regarding CKD, this is a very common type of disease, which, if detected in time, can be managed through periodic therapies. Thanks to the University of California, Irvine (UCI), which made public the database known as UCI CKD [64] (containing 24 characteristics, derived from patient history and diagnostic tests, plus information regarding the presence or absence of CKD), many studies have been developed to diagnose CKD. Since this database was made public, various studies have used it to test multiple different types of solutions, obtaining increasingly impressive results for accuracy (63–100%) [65,66,67,68,69,71,72,74,76], AUC (0.995) [70], and F1 score (100%) [73]. Being the only public database available for this pathology, the research has been mainly focused on the analysis of numerical features; this is also due to the fact that patients suffering from CKD, or otherwise at risk, cannot undergo all the existing diagnostic imaging techniques. In this case, techniques that require the use of radiation, such as CT, are strongly discouraged, because they can easily worsen the patients’ condition. Therefore, imaging techniques such as US, used in [26] with 82% accuracy in predicting the stage of CKD, and MRI are preferred. The latter has been shown to have the ability to allow assessment of both renal function and structure [120]. Major future developments may shift in this direction and focus on the development of methods that take advantage of MRI to be able to determine CKD.

If radiation imaging techniques cannot be used to determine CKD, the same is not true for detecting and analyzing kidney stones. In particular, for kidney stones, it is possible to use not only CT but also low-dose CT (LDCT), which exposes the patient to approximately five times less radiation than regular CT [121]. The independence of the dosage used to acquire CT is demonstrated in several studies: in [81], ML techniques are applied to process LDCT and CT and identify the composition of a kidney stone, achieving 86% accuracy for both assays used; in [82], LDCT is analyzed to differentiate between kidney stones and phleboliths in patients with acute flank pain, with 85.1% accuracy. The applicability of these methods ensures that low-dose radiation CT acquisitions can be used for the detection of a kidney stone, reducing any risks associated with the radiation exposure of normal CT. In addition to the detection and analysis of kidney stones, researchers are also studying the prediction of success in removing a kidney stone. Successful selection of the most appropriate method can lead to a higher rate of kidney stone clearance, lower risk of associated morbidities, higher probability of survival, faster recovery, and lower overall cost of care [122]. Depending on the procedure chosen [84,85], and for the prediction of stone removal, there is 60% accuracy [84] for predicting success after the first treatment, and 87.9% for predicting success when a shock wave is used for kidney stone clearance [85]. Accuracies ranging from 81% to 98.2% have been obtained for predicting a patient’s condition and possible complications following renal stone removal [83].

Since glomerular disease is a condition that worsens over time, the machine learning techniques implemented are primarily focused on predicting the prognosis of the condition and identifying the consequences caused by the presence of the disease [87,88,89,92,93,94,95,96,97,98]. The most common glomerular disease prevalent in the world is Immunoglobulin A Nephropathy (IgAN) [123]. IgAN is caused by renal dysfunction and can be diagnosed by diagnostic imaging of the kidney, particularly immunofluorescence imaging. Some researchers have focused on diagnosing IgAN from diagnostic images with different resolutions, with an accuracy of at least 80% [92] and an accuracy of 80.27% [95], using only clinical and laboratory analysis data. Around 30–40% of IgAN patients carry the risk of the disease degenerating into ESRD (end-stage renal disease) [93]; for this reason, some research tries to predict this degeneration to allow the efforts of physicians to focus mainly on patients who are more at risk, as, for example, in [93], where it predicts the degeneration of the disease in the next 5 years, with AUC of 0.82, and after 10 years with AUC of 0.89, and as in [94], with 79.8% accuracy. Another particular type of glomerular disease is caused by diabetes. Since diabetes is very common, it is very important to prevent its degeneration into diabetes kidney disease, and in [87,88,89], the authors focus precisely on this aspect by creating algorithms that can predict the prognosis, with an accuracy of 83.5–94%.

Regarding the literature inherent to renal transplantation, it is possible to identify three possible applications of AI [123]: (i) diagnosis, using AI to diagnose the level of transplant risk by detecting parameters associated with renal transplant rejections, and identifying abnormal patterns within them, as in [104], with 68.4% accuracy, and in [106]; (ii) prescription, using AI to prescribe postoperative therapies [124] to prevent complications or rejection, or to prescribe diets that may improve quality of life after renal transplantation [125]; (iii) prediction, using AI to predict mortality, and possible rejection, as in [102], with 73.8% specificity and 88.2% sensitivity; in [103], with 56% accuracy over a 3-year timeframe from possible rejection, and in [105], with 85% accuracy. It is important to note that for this specific task, the main limitation for the application of AI is given by the fact that the type of database is very patient-specific [103,104,105,106], as the values are highly dependent on both the recipient and the donor(s) available, resulting in a limitation that makes it difficult to generalize the solutions devised [126].

Before concluding, we believe that it is also necessary to analyze the ML algorithms used in nephrology, to address a possible reader interested in a specific type of algorithm rather than another, depending on the type of application that they would like to achieve. First of all, it is possible to notice that all the ML algorithms used are based on the use of supervised learning techniques. This is mainly due to the fact that the realized tasks are formulated and viewed in the form of classification problems. In particular, with regard to the research identified in this work, in Table 10, all the methods used have been grouped by algorithm type.

From the table, it can be observed that the simplest and most common classification algorithms, such as random forest and support vector machine, and ensemble algorithms, such as gradient boosting machine, are the most used in these types of studies. However, more complex ML algorithms, such as artificial neural network, and deep neural networks, such as convolutional neural network, autoencoder, and more sophisticated approaches based not only on feature or image analysis, but also on natural language processing and the temporal evolution of features (temporal-based approaches, e.g., recursive neural network) are not missing. This could be due to the lack of very large public databases that would allow better use of the more complex ML techniques [127].

It is also possible to note that the methods applied by the authors differ mainly with respect to the type of the used data and the techniques of analysis and data processing. In particular, in cases where the database is composed exclusively of numerical features, derived from patients’ medical records, classifiers such as support vector machine, random forest, and artificial neural network are the most frequently applied. Whenever diagnostic images are present, instead, the type of ML technique varies according to the preprocessing applied to the data. In the case of minimal or null preprocessing, techniques such as convolutional neural network are used, in which the model directly analyzes the image and finds the most relevant features in order to classify it. Instead, when algorithms are used for the extraction of radiomic features from specific anatomical regions, algorithms generally applied to numerical features are used; in particular, ensemble algorithms are exploited, which typically, in these cases, guarantee a better result in terms of metrics.

Finally, for the evaluation of algorithms’ performance, the authors feel that it could be misleading to compare methods applied to the same objective based on the values obtained from the evaluated metrics computed with different data. However, it is possible offer some considerations about the various metrics used, in order to understand in which cases some metrics are used instead of others. Since the most commonly used metrics are accuracy and AUC, we consider it appropriate to briefly discuss what the differences are: accuracy is a metric that represents the ratio of the number of correctly predicted samples to the total number of samples present; AUC, on the other hand, represents the area under the receiver operating characteristic (ROC) curve that shows, for different probability thresholds, the relationship between the false positive rate (ratio of the number of false positives to the total number of negative cases) and the true positive rate (ratio of the number of true positives to the total number of positive cases). Looking at the two definitions, it may be deduced that the accuracy is a more intuitive metric and therefore more frequently used, but its simplicity has drawbacks, since it cannot be used in all cases—for example, in the case of unbalanced datasets, where it is preferable to use metrics such as the F1 score or AUC, or in case it is desired to take into account the probability associated with the various classes predicted, in which case only AUC takes this aspect into account. With the above in mind, the use of AUC is strongly recommended as it encapsulates increasingly confident information than accuracy alone.

Despite the limits of this work, given the continuous evolution of research in this area, based on what has been analyzed so far, it is possible to conclude that, given the many existing applications of ML in nephrology, AI has great potential and versatility in this field. An example of a possible application for kidney image analysis can be based on the combination of the multiple methodologies that currently exist, such as the use of deep learning to detect kidneys and tumors [38,39,40], followed by the use of other machine learning techniques to classify the nature and/or severity of tumors, or the presence of any kidney disease and/or other possible masses. However, this does not mean that limitations are not still present. Most of the studies identified end before moving to a clinical trial, remaining only single-center retrospective studies, reducing their external validity [128,129]. Consequently, the main and most urgent gap that should be addressed as soon as possible is that of the public availability of data; this will not only allow studies to be compared with each other but will ensure that there are improvements in nephrology itself [2]. To this end, the guidelines for conducting clinical trials in nephrology, reported at the Kidney Disease—Improving Global Outcomes (KDIGO) conference, could be followed [126].

## 6. Conclusions

In this work, fifty-nine, from a total of 224, studies concerning the application of ML techniques for the segmentation, prediction, and classification of renal diseases were analyzed. First, the studies were divided, analyzed, and presented based on the addressed pathology and the main goal of the research. Then, the existent datasets were analyzed in terms of data typology, size, and public availability; the main concept derived from this analysis is the importance of a large dataset and public availability to allow research to go as far as possible for a specific objective. Finally, the various pathologies were discussed in terms of what does not exist and what can be done to achieve further developments in this specific sector. In conclusion, from the analysis of the literature, it can also be noted how the introduction of modern ML techniques in the nephrological field allows the achievement goals not obtainable with traditional techniques, such as speeding up and automating CT segmentation processes, the possibility to perform non-invasive and reliable diagnosis, and to create predictive models—for example, to evaluate surgical or transplant outcomes and create predictive models to monitor patient’s parameters in order to act promptly.

Among all the works analyzed, it can be seen that the practical purposes of the use of AI in urology range from the diagnosis of a disease, to the analysis of diagnostic images, to the prediction of prognosis, etc., and generally aim to aid doctors in making more accurate decisions, without attempting, in any way, to replace them [130,131,132,133]. The physician’s attendance remains essential both from a human point of view, in establishing a deep doctor–patient bond of trust that can improve the success of any therapies and treatments [134], and from an ethical and accountable point of view for diagnoses [135].

## Figures and Tables

**Table 1:** Renal mass research.

Paper	Objective	Method	Database	Results	Year
[27]	Malignant renal cyst prediction	Bayesian classifier	[27]	AUC 0.96	2009
[28]	Identify malignant renal masses	Statistical relational learning—RFGB: relational functional gradient boosting	[28]	Accuracy 82%	2018
[29]	Differentiate between malignant and benign masses	CT texture analysis with random forest	[29]	Accuracy 90.5% AUC 0.915	2020
[30]	Diagnose ccRCC	WEKA with and without SMOTE	[41]	AUC contour-focused 0.865–0.984 AUC margin shrinkage 0.745–0.887	2019
[31]	Diagnose ccRCC	Pyradiomics and random forest	[41]	Accuracy 84.6% Sensitivity 90.4% Specificity 78.8% Precision 81%	2020
[32]	Diagnose ccRCC	Radiomics and CatBoost	[32,41]	MR accuracy 73% internal 74% external CT accuracy 79% internal 69% external	2020
[33]	Diagnose ccRCC	MaZda and WEKA toolkit	[33]	Accuracy 85.1%	2018
[34]	Diagnose ccRCC	Proteomics-based random forest and imaging-based VGG16	[41]	Proteomics accuracy 98% image accuracy 83% validation, 95% testing set	2019
[35]	Differentiate between kidney chromophobe renal cell carcinoma and oncocytoma	Linear SVM	[35]	Accuracy 80%	2016
[36]	Classify papillary renal cell carcinoma stages	Feature extraction and random forest	[42,43]	Accuracy 88.5%	2018
[38]	Kidney and tumor segmentation	3D U-Net	[44]	Mean Kidney Tumor Dice 0.9168	2019
[39]	Kidney and tumor segmentation	Cascade 3D U-Net	[44]	Mean Kidney Tumor Dice 0.9064	2019
[40]	Kidney and tumor segmentation	Multi-resolution 3D V-Net	[44]	Mean Kidney Tumor Dice 0.8815	2019

**Table 2:** AKI research.

Paper	Objective	Method	Database	Results	Year
[45]	Predict AKI in adult and children	Boruta [56] (selection algorithm) + random forest	[57,58]	AUC 0.796	2018
[46]	Predict AKI in adult and children	Gradient boosted machine	[59]	AUC 0.85	2021
[47]	Predict AKI	Gradient boosted machine	[47]	AUC 0.76	2021
[48]	Predict AKI in burn patients	K-NN	[48]	Accuracy 97%	2019
[50]	Predict AKI	Lasso + logistic regression	[60]	AUC 0.79 AUC 0.82 [*p* < 0.001]	2019
[51]	Predict AKI	RF + XGboost	[51]	AUC 0.843	2020
[53]	Predict AKI	Streams	[53]	Accuracy 56% in 48 h Accuracy 84% in 30 d Accuracy 90% in 90 d	2019
[54]	Prediction of AKI from blood test	Feature selection + random forest	[54]	AUC 0.881 in 30 d	2021
[55]	Predict AKI	Gradient boosting tree-based machines	[61]	AUC 76% in 48 h AUC 81% stage 2 AUC 87% stage 3	2020

**Table 3:** CKD research.

Paper	Objective	Method	Database	Results	Year
[26]	Diagnose CKD based on patient stage	Support vector machine—SVM	[26]	Accuracy 82% on 2 stages Accuracy 67.21% on 3 stages Accuracy 51% on 5 stages	2014
[65]	CKD diagnosis	Random forest	[64]	Accuracy 99.3%	2016
[66]	CKD diagnosis	Decision tree C4.5	[64]	Accuracy 63%	2016
[67]	CKD diagnosis	SVM	[64]	Accuracy 98.3%	2016
[68]	CKD diagnosis	k-NN with CFS and AdaBoost	[64]	Accuracy 98.1%	2017
[69]	CKD diagnosis	Random forest	[64]	Accuracy 100%	2017
[70]	CKD diagnosis	RPART	[64]	AUC 0.995 Sensitivity 0.9897 Specificity 1	2018
[71]	CKD diagnosis	PSODP + DL-RNN	[64]	Accuracy 99.5%	2018
[72]	CKD diagnosis	PNN [77]	[64]	Accuracy 96.7%	2019
[73]	CKD diagnosis	RFE and Random Forest	[64]	F1 score 100%	2021
[74]	Predict diet plan for CKD patients	Multiclass Decision forest	[64]	Accuracy 99.17%	2017
[76]	Predict hemoglobin levels in CKD patients	Extraction rule—Re-RX + J48graft	[64]	Accuracy 95.18%	2019

**Table 4:** Kidney stone research.

Paper	Objective	Method	Database	Results	Year
[81]	Renal stone detection	Segmentation + ANN	[81]	Accuracy 86%	2019
[82]	Renal stones vs. phleboliths	Radiomics + AdaBoost classifier	[82]	Accuracy 85.1%	2019
[83]	Kidney stone removal, prediction of postoperative variables	ANN	[83]	Accuracy 81–98.2%	2017
[84]	Predict stone-free status after the first treatment	Feature extraction + sequential forward selection + multiple classifier scheme	[84]	Accuracy 60%	2019
[85]	Stone-free prediction	Light gradient boosting method	[85]	Accuracy 87.9%	2020

**Table 5:** Glomerular disease research.

Paper	Objective	Method	Database	Results	Year
[87]	Predict diabetic kidney disease	SVM radial	[87]	Accuracy 94%	2013
[88]	Predict diabetic kidney disease	Unbalanced random forest	[88]	Accuracy 83.8%	2018
[89]	Predict diabetic kidney disease	Knime + WEKA	[89]	Accuracy 83.5%	2019
[92]	Resolution image-based renal pathology	Convolutional neural network	[92]	Accuracy > 80%	2021
[93]	Predict ESKD in patients with IgAN	ANN	[93]	AUC 0.82 with 5-year follow-up AUC 0.89 with 10-year follow-up	2021
[94]	Predict deterioration of kidney function in IgAN patients	SVM	[94]	Accuracy 79.8%	2021
[95]	Diagnose glomerular disease	Disjunctive least generalization—DLG algorithm	[95]	Accuracy 81.26–96.5%	1992
[96]	Detect pathogenic and non-pathogenic glomerulus and tubulus	RatSnake—ML automatic segmentation	[96]	Accuracy 94.7%	2014
[97]	Diagnose glomerular disease	Decision tree with J48 algorithm	[97]	Accuracy 89.47%	2021
[98]	Predict weight of children in renal dialysis	ANN	[98]	Mean difference 0.497	2018

**Table 6:** Kidney transplant research.

Paper	Objective	Method	Database	Results	Year
[102]	Predict transplant failure probability	Decision tree	[102]	Specificity 73.8% Sensitivity 88.2%	2010
[103]	Predict post- transplant survivability	Bayesian belief network	[103]	Accuracy 52% after 1 year Accuracy 56% after 3 years	2012
[104]	Classify risk levels for kidney graft survival after transplant	ElasticNet + Bayesian belief network	[107]	Accuracy 68.4%	2018
[105]	Predict early transplant rejection	Decision tree and random forest	[105]	Accuracy 85%	2019
[106]	Predict kidney transplantation compatibility Predict renal function worsening 1 year after transplant	Elderly KTbot	[106]	Precision 90% Sensitivity 71% F1 score 0.79	2020

**Table 7:** Other renal diseases research.

Paper	Objective	Method	Database	Results	Year
[108]	Predict hemoglobin in patients with kidney disfunction	Data merging + clustering + ensemble of classifiers	[108]	Mean absolute error 0.662—Italy, mean absolute error 0.673—Spain	2014
[110]	Recommend renal biopsy	Tokenization + NLP machine learning classifier	[110]	Accuracy 83.5% Precision 80.6%	2019
[111]	Prediction of 1-year survival in hemodialysis patients	Ensemble artificial intelligence model	[111]	Accuracy 94.8%	2020
[112]	Detect kidney and liver tissue for hydronephrosis patient	Homodyne-K feature extraction + random forest	[112]	Accuracy 94%	2015
[113]	Predict risk stratification of renal function deterioration based on eGFR threshold	Multitask temporal-based classifier	[113]	Specificity 0.828 with 10% threshold Specificity 0.786 with 20% threshold	2015

**Table 8:** Diagnostic image databases.

Database	Number of Patients	Description	Year (First Use/Published)	Open Access
[27]	93	Patients’ MDCT. Patients with complicated cysts: cyst with at least one focus of septa, a solid nodule, and any calcification or wall thickening on MDCT	2009	No
[28]	150	Patients’ CT. 100 malignant tumors: 70 clear cell renal cell carcinoma (ccRCC), 20 papillary renal cell carcinoma (pRCC), and 10 chromophobe renal cell carcinoma (chRCC); 50 benign tumors: 20 lipid-poor angiomyolipoma (lpAML), 30 renal oncocytoma	2018	No
[29]	79	84 renal masses: 63 malignant (25 clear cell RCC, 23 papillary cell RCC, 15 chromophobe RCC), 21 benign (10 oncocytomas, 11 fat-poor angiomyolipomas)	2020	No
[32]	440	440 MRI and CT of patients with ccRCC	2020	No
[33]	54	Patients’ CT. All patients have ccRCC.	2019	No
[41]	216	216 proteomics data and 783 slide images (524 tumoral)	2018	Yes
[44]	300	CT of patients with one or more kidney tumors. Segmentation of kidneys and tumors.	2019	Yes
[26]	188	The database is composed of 40, 16, 38, 60, 28, and 6 entries for healthy, stage 1, 2, 3, 4, 5, respectively. These images are obtained from 35 observers taken at different times. The kidney ultrasonic images are segmented and annotated into three regions of interest (ROIs)	2014	No
[81]	200	200 kidney stones harvested from nondestructive stone extraction at three different sites. Stone size was measured using a digital caliper	2020	No
[82]	412	LDCT of 235 kidney stones and 224 phleboliths	2019	No
[83]	254	Preoperative abdominopelvic ultrasound and intravenous urography or CT scan of PCNL patients.	2017	No
[112]	9	This dataset contains the 3D US abdominal images from 9 pediatric patients with hydronephrosis	2015	No
[96]	1321	Biopsy images of pathogenic (338) and nonpathogenic (396) glomerulus and some of pathogenic (338) and nonpathogenic (248) tubulus	2014	No
[97]	584	Renal biopsy reports, each of 4 or 5 slides with different stains, for each case: clinical and laboratory data, diagnostic hypothesis, histological biopsy study, histological report of glomerular disease	2021	No
[92]	422	Renal immunofluorescent images obtained by fluorescence microscopes relative to a renal biopsy of 162 patients with IgAN and 260 without	2021	No
[35]	24	24 unstained deparaffinized formalin-fixed kidney tissue sections of chRCC and oncocytoma, 12 of each type	2016	No

**Table 9:** Numerical databases.

Database	Number of Patients	Description	Year (First Use/Published)	Open Access
[42]	260	Tumor RNASeq and pathological stage (I, II, III, and IV): Stage I—172, Stage II—22, Stage III—51, and Stage IV—15.	2010	Yes
[43]	34	This dataset was obtained using Affymetrix HGU133 Plus 2.0 array platform and includes 19 and 15 samples in early (excellent survival) and late (poor survival) stages of PRCC.	2005	Yes
[57]	269,999	6.1% of patients in the dataset had a clinical deterioration event: 424 cardiac arrests, 13,188 intensive care unit (ICU) transfers, and 2840 deaths on the wards. For each patient, there are a total of 29 features.	2014	No
[59]	108,441	Australian and New Zealand Society of Cardiac and Thoracic Surgeons Database registry recorded 110,342 cardiac surgery events in 108,441 unique patients.	2018	No
[47]	780	Medical data collected by natural language process module from EMRs including demographic data, daily documentation, laboratory and imaging results, anesthesia records, medications, interventions, and diagnosis. TRIPOD guidelines were followed.	2021	No
[48]	50	Serial creatinine testing of patients with ≥20% total body surface area (TBSA) burns at risk for AKI. AKI was defined using the Kidney Disease: Improving Global Outcomes (KDIGO) criteria.	2019	No
[61]	153,821	153,821 patients from 6 different sites. Each patient had a mean of 67 (SD = 46) clinical facts per day.	2020	No (only in USA)
[51]	671	Information related to demographic characteristics, clinical condition, preoperative biochemistry data, preoperative medication, and intraoperative time-series hemodynamic features (systolic blood pressure (SBP), diastolic blood pressure (DBP), mean arterial blood pressure (MAP), and heart rate (HR)) from electronic medical records and records on intraoperative variables.	2020	No
[54]	51,869	618,719 blood test occurrences for 51,869 distinct patients.	2021	No
[64]	400	The CKD dataset was collected from 400 patients from the University of California, Irvine Machine Learning Repository.	2015	Yes
[84]	254	This dataset includes information on preoperative, intraoperative, and postoperative parameters from 254 patients who underwent kidney surgery.	2019	No
[93]	1015	The variables contained per IgAN patient are age, sex, hypertension, serum creatinine, daily proteinuria, kidney biopsy, therapy—RASBs or corticosteroids. The primary outcome is ESRD, dialysis, or transplantation.	2020	No
[94]	80	Features of 80 IgAN patients: secondary IgA deposition, eGFR, MEST-C scores.	2021	No
[95]	284	38 features for each patient and biopsy diagnosis.	1992	No
[102]	194	Features for each patient: age, sex, time in dialysis, donor type, donor age, HLA mismatches, delayed graft function, acute rejection episode, and chronic allograft nephropathy.	2010	No
[103]	7348	A total of 793 pre- and post-transplant variables per patient.	2004	No (only in USA)
[58]	6564	First 12 h of 6564 HER from critically ill children admitted to a pediatric ICU without evidence of AKI; 4% of the patients developed AKI by 72 h.	2016	No
[53]	2642	The dataset contains the data relative to 1781 patients pre-implementation and 861 patients post-implementation of a digital intervention system, with the relative alert severity.	2019	No
[85]	358	This dataset includes 42 features including the two target variables, stone-free and one-session success, for all 358 cases. The number of cases with stone-free and one-session success was 253 (70.7%) and 154 (43.0%).	2020	No
[60]	1250	Several serum markers per patient undergoing angiography as clinical standard care.	2015	No (only in USA)
[105]	80	80 patients who received HLA-incompatible renal allografts; 14 features measured before transplantation.	2019	No
[106]	118	Medical records of 18 elderly and 100 younger patients.	2020	No
[87]	1386	Anthropometric measurements and blood pressure (BP), drug use and past medical history, physical assessment for retinopathy, sensory neuropathy, and peripheral arterial disease. eGFR calculated using the Chinese-modified Modification of Diet in Renal Disease equation.	2013	No
[88]	1000	1000 T2DM patients’ data collected by the IRCCS (Istituto di Ricovero e Cura a Carattere Scientifico) of the Hospital of Pavia.	2018	No
[89]	~32,000	Diabetes of type 2 patients with a 24-month analysis window.	2019	No
[110]	3149	This dataset contains a total of 3149 admission notes from the nephrology department. For the ground truth, there are recommendations given by physicians in first-day progress notes.	2019	No
[113]	6435	Electronic health records of patients with hypertension, diabetes, or both.	2015	No
[107]	~31,000	United Network for Organ Sharing, a private, non-profit (UNOS) dataset including information on all kidney waiting-list registrations and transplants that had been recorded in the U.S.	2014	No (only in USA)
[108]	13,011	125 features from dialysis clinical practice of 13,011 patients.	2014	No
[98]	14	ESRD patients on chronic hemodialysis or hemodiafiltration weighing 20 kg or more.	2018	No
[111]	79,860	Various features for each patient are presented with the relative risk score based on mass, serum albumin level, cholesterol level, and creatinine.	2020	No

**Table 10:** Searches grouped by type of ML algorithm applied.

Method—ML Algorithm (Based)	Authors	Year
Bayesian classifier	[27]	2009
	[103]	2012
	[104]	2018
Logistic regression	[50]	2019
	[106]	2020
Decision tree	[66]	2016
	[97]	2021
	[102]	2010
Random forest	[112]	2015
	[65]	2016
	[69]	2017
	[45]	2018
	[36]	2018
	[34]	2019
	[105]	2019
	[29]	2020
	[31]	2020
	[73]	2021
	[54]	2021
	[88]	2018
SVM	[87]	2013
	[26]	2014
	[35]	2016
	[67]	2016
	[94]	2021
ANN	[81]	2019
	[83]	2017
	[98]	2018
	[93]	2021
Ensemble of classifiers	[84]	2019
	[111]	2020
	[108]	2014
	[68]	2017
	[48]	2019
	[82]	2019
	[76]	2019
	[33]	2018
	[89]	2019
	[30]	2019
	[28]	2018
	[46]	2021
	[47]	2021
	[55]	2020
	[85]	2020
	[51]	2020
	[32]	2020
DNN	[96]	2014
	[113]	2015
	[110]	2019
	[53]	2020
	[95]	1992
	[70]	2018
	[71]	2018
	[72]	2019
	[34]	2019
	[92]	2021
	[38]	2019
	[39]	2019
	[40]	2019
